# Does CSR affect the cost of equity capital: Empirical evidence from the targeted poverty alleviation of listed companies in China

**DOI:** 10.1371/journal.pone.0227952

**Published:** 2020-02-07

**Authors:** Yuting Yi, Bangsheng Xie, Lixue Zhou, Yuanzhu Wei

**Affiliations:** 1 College of Management, Fujian Agriculture and Forestry University, Fuzhou, China; 2 Ningde Normal University, Ningde, China; The Bucharest University of Economic Studies, ROMANIA

## Abstract

Social responsibility fulfillment helps modern enterprises achieve sustainable development. Based on empirical data on China's A-share listed companies in 2013–2016, this paper examines the impact of corporate social responsibility performance on a company's financing costs from the perspective of targeted poverty alleviation. Specifically, we find that enterprises’ engagement in poverty alleviation social responsibility helps to reduce the cost of equity capital. The result is robust to using alternative indicators of the cost of equity capital, propensity score matching method, change model and sample removed financial sector. Furthermore, we find that the negative relationship between enterprises’ engagement in poverty relief and the cost of equity capital is mainly concentrated in private enterprises and in the central and eastern regions of China. Moreover, the negative relationship mainly exists after China’s listed companies were forced to disclose information on poverty alleviation. This paper also finds that institutional investors' shareholding plays a partial mediating role in this reduction effect and that enterprises’ poverty alleviation efforts help companies improve their financial performance and firm value. This study enriches the relevant literature on corporate social responsibility and the cost of equity capital and has reference value for corporate sustainable development. It also provides a theoretical basis for corporate poverty alleviation work in developing countries and the economic results of CSR.

## Introduction

Corporate sustainability is closely related to corporate social responsibility (CSR) [1,2]. Since corporate sustainability is a source of competitiveness, companies need good sustainability management, which includes efforts to improve their environmental or social behavior, namely, the fulfillment of social responsibility [1]. CSR is the means of adapting the current sustainable development framework in the process of corporate sustainable development. Studying CSR helps provide an understanding of the way in which corporate sustainability can be achieved [2]. Previous studies on CSR have focused on the influencing factors or economic consequences of corporate environmental social responsibility or charitable social responsibility [3,4,5]. However, anti-poverty issues also require attention. Alleviating poverty in various countries has always been one of the UN's Millennium Development Goals, and it is related to the sustainable development of human society. International organizations such as the World Bank and the United Nations also hope that private sectors such as enterprises can play a role in the realization of human anti-poverty goals, and thus corporate poverty alleviation can help achieve sustainable development goals [6].

China has made huge strides in its battle against poverty as it has transformed into one of the most dynamic economies in the world [7]. To reach the country's target of eradicating poverty in rural areas and eliminating regional poverty by 2020, the Communist Party of China (CPC) included poverty alleviation in the "five-sphere integrated plan" and "four-pronged comprehensive" strategy, putting it in an important position in country governance. To encourage capital market enterprises to participate in poverty alleviation, "Opinions of the China Securities Regulatory Commission on the Role of Capital Markets in Serving the Country in Poverty Alleviation" proposed to give priority to supporting enterprises in poverty-stricken areas to use capital market resources, broaden direct financing channels, improve financing efficiency, and reduce financing costs. At the end of 2016, the Shanghai and Shenzhen Stock Exchanges comprehensively refined the information disclosure requirements for targeted poverty alleviation and mandated that listed companies report their involvement in poverty alleviation in a specified detailed table in their annual reports. With the government's emphasis on poverty eradication, the number of Chinese A-share listed companies involved in targeted poverty alleviation has gradually increased (according to manually collected data, the number of companies engaged in poverty alleviation efforts increased from 48 in 2013 to 754 in 2017). Given this context, why do companies participate in targeted poverty alleviation, and what are the economic consequences? How do corporate stakeholders view companies that engage in poverty relief? Does poverty alleviation participation affect corporate financing costs? These issues need to be answered urgently in the new era of poverty alleviation. Therefore, this paper chooses targeted poverty alleviation data in the context of poverty alleviation to study the economic consequences of CSR from a new perspective.

Many studies have discussed the relationship between CSR and firm value [8–17]. However, little attention has been paid to financing costs, such as the cost of capital. As the cost of equity capital reflects investors' risk expectations for the company [18], it is closely related to corporate finance and sustainable development [18,19]. Research indicates that information disclosure, corporate governance, taxation, and audit quality, among other factors, can influence the cost of equity capital [20–25], and some scholars believe that social responsibility report disclosure and assurance can alleviate information asymmetry, reduce investor supervision costs, and thus reduce the cost of equity capital [26,20]. However, no study has discussed the impact on the cost of equity capital from the perspective of enterprises’ involvement in poverty alleviation. Therefore, this paper chooses the cost of equity capital to explore the impact of CSR on financing costs. Then, for capital market investors, this study examines whether the effort of listed companies' targeted poverty alleviation is a signal that increases the value of enterprises or a kind of agency cost that increases risk expectations and, in turn, affects the cost of equity capital? We manually collect data about poverty alleviation for Chinese enterprises from 2013 to 2016 to test this issue.

This study is the first empirical test of the economic consequences of enterprises’ targeted poverty alleviation and provides positive empirical evidence from the capital market for China's ongoing poverty alleviation and a reference for CSR in developing countries. The previous literature mainly focused on the field of environmental responsibility [3,4]; by contrast, this paper is based on the background of China's poverty alleviation in recent years, further complementing the study of the economic consequences of CSR and research on the cost of equity capital and providing a new research perspective. In addition, this paper further verifies the impact of institutional investors on corporate finance and development in the capital market and provides preliminary empirical evidence for the role of institutional investors in CSR.

The rest of this paper is organized as follows. The second section presents the background and relevant literature and develops the hypotheses. The third section describes the materials and methods. The fourth section reports the empirical results. The last section concludes the paper and offers suggestions and directions for future research.

## Literature review and theoretical background

### The cost of equity capital

Most of the literature on the cost of equity capital focuses on the impact of information disclosure and corporate governance [21,27,22]. For example, Botosan [21] documents that the level of information disclosure is conducive to alleviating adverse selection and reducing the perceived risk of investors, thereby reducing the company's cost of equity capital. Chen et al. [22] suggest that corporate governance, as a mechanism for protection external investors from infringement by company insiders, can not only reduce the market risk of investors but also reduce the supervision cost and information asymmetry of external investors, thus reducing the risk premium required by investors, that is, the cost of equity capital. Some scholars have also paid attention to the impact of corporate taxation, audit quality, investor protection and internal control on the cost of equity capital [23–25,28]. In contrast to the extensive literature on the cost of equity capital, there are few studies on the relationship between CSR and the cost of equity capital, and few scholars have studied the impact of CSR disclosure or CSR reporting verification on the cost of equity capital [29,30,20,26]. El Ghoul et al. [30] provide theoretical evidence that environmental social responsibility can reduce the possibility and cost of negative events, thereby reducing investors' risk expectations. Therefore, the increase in investor stock holding demand and the reduction in risk expectations reduce the cost of capital. Dhaliwal et al. [20] show that social responsibility information can increase institutional investors' shareholding and improve analysts' prediction accuracy, thus alleviating information asymmetry and ultimately reducing the cost of equity capital. These studies examine the relationship between CSR and the cost of equity capital, and the present study fills a gap in the literature by providing the first analysis of the impact of enterprises’ poverty relief work on the cost of equity capital.

### The economic consequences of CSR

CSR is defined as the notion that corporations have an obligation to constituent groups in society other than stockholders and beyond that prescribed by law or union contract, indicating that a stake may go beyond mere ownership [31]. In terms of the economic consequences of CSR, there are two opposing views, namely, risk reduction and overinvestment. The risk-reduction perspective posits that CSR reduces corporate risks and enhances corporate value. Extant studies have found that CSR performance can bring ethical capital to companies and help to enhance shareholder value and returns [5,32], and other research suggests that CSR can enhance corporate governance and supervision, reduce insider trading motives, reduce information asymmetry, improve earnings quality and reduce the risk of stock price collapse [33–37,10], supporting the idea of risk reduction and reduced corporate cost of equity capital [20,26,38,39]. For example, Zolotoy et al. [38] state that charitable contributions can alleviate agency problems and reduce investor risk expectations, thereby reducing the cost of capital. Dhaliwal et al. [26] demonstrate that CSR disclosure can reduce information asymmetry and increase information transparency, reducing the cost of equity capital. Cheng et al. [40] conclude that social responsibility allows more stakeholders to understand corporate affairs, promotes information exchange between enterprises and stakeholders, and thus eases financing constraints. In contrast, the overinvestment perspective regards social responsibility as a corporate excessive investment behavior that will increase the risk of the enterprise and damage its value. For example, Bhandari and Javakhadze [41] find that CSR investment consumes resources needed for projects that can increase corporate value, thus reducing the efficiency of corporate investment, increasing the agency cost of the company, and ultimately damaging shareholder value. Hemingway and Maclagan [14] hold the view that personal factors of management may influence decision-making related to social responsibility. If the fulfillment of CSR is due to a motive to conceal the company's improper behavior rather than moral factors, firm risks may increase. For example, a study has already found that management regards CSR as a tool to conceal bad news [15]. According to the literature above, we can conclude that existing research on CSR mainly focuses on specific areas such as environment and charity and rarely studies the economic consequences of poverty alleviation.

### Hypothesis development

Resource-based theory holds the view that CSR can help companies obtain the necessary tangible or intangible resources to form a long-term and nonreplicable competitive advantage [8,42] and that the acquisition of competitive advantage can enhance future value, reduce the various risks faced by the company, and thus affect the cost of equity capital. Overall, this study posits that corporations' engagement in poverty relief mainly affects the cost of equity capital from the following three aspects. First, it helps to reduce information asymmetry. The fulfillment of CSR promotes communication between shareholders and management, increases information transparency, and reduces information asymmetry between enterprises and investors [11]. Kim et al. [36] state that CSR performance reflects management and corporate ethics and a high level of accounting information quality. In addition, Cheng et al. [40] suggest that CSR fulfillment enables more stakeholders to participate in corporate affairs and increases information transparency. Therefore, as an important part of CSR, enterprises' participation in targeted poverty alleviation can send external investors a good signal for corporate development and reduce information asymmetry with investors. Second, it can enhance corporate governance and supervision and reduce investor supervision costs. The literature finds that CSR fulfillment provides employees a sense of identity, a belief that the company is more fair, and a sense of belonging; as a result, they are more likely to work harder and abandon bad behaviors [43]. In addition, undertaking corporate social responsibility can attract high-quality employees and increase the competitive advantage in human capital [42], which may enhance corporate development and reduce the cost of supervision of external investors. Dhaliwal et al. [20] document that companies with good social responsibility performance can attract institutional investors to hold shares and increase analyst forecast accuracy. These findings show that CSR performance can enhance corporate governance, reduce the supervision costs of other investors, and thus reduce the necessary return rate required by investors. Third, it may reduce the risk expectations of investors. CSR performance can bring ethical capital to the company and generate a reputational insurance effect, while reputational insurance can reduce the likelihood and cost of negative events and may reduce the negative impact on firm value when a negative event occurs, thus reducing corporate risk [44]. In addition, CSR practices can increase consumer purchase intentions and sales revenue [45,13], strengthen government-enterprise relations and build government trust in the enterprise [46], which helps companies to enhance firm value and reduce political risks. Accordingly, expectations of future value enhancement and risk reduction also make investors more willing to hold corporate stocks in the hope of achieving greater returns in the future [47], and the risk-sharing of increased shareholding demand will also reduce the cost of equity capital [30].

On the other hand, enterprises’ efforts in helping people out of poverty may also increase corporate risk and increase the return rate required by shareholders. First, decisions related to CSR are susceptible to management's personal factors [15]. Chih et al. [48] believe that the motivation of management to make CSR decisions is not moral reasons but rent-seeking and other purposes, such as providing themselves with an opportunity to manipulate profit and achieve personal benefits (i.e., compensation and bonuses). A previous provides direct evidence that social responsibility does not reduce the degree of information asymmetry and is used by management as a tool to conceal bad news, dissipate public attention and disguise management failure or other behaviors, ultimately increasing earnings management [16]. Second, Hubbard et al.’s [49] findings imply that in companies with poor performance, excessive CSR investment may even lead to investors suspicions about the CEO's ability, increasing the risk of CEO dismissal, i.e., increasing the possibility of staff turnover risk. Finally, fulfilling social responsibilities may sacrifice resources for other positive investment projects and divert managers’ attention, thereby reducing capital allocation efficiency, sacrificing shareholders' interests and undermining corporate value [41]. Therefore, the involvement of enterprises in poverty alleviation may also be regarded by investors as a tool for concealing bad news such as failure of internal investment projects or a decline in operating performance, which increases the uncertainty and risk of shareholder investment, causing investors to increase the cost of equity capital to compensate for the increased risk. Accordingly, given the arguments above, we propose the following hypothesis.

**Hypothesis:** Firms that participate in targeted poverty alleviation lower the cost of equity capital compared with firms that do not.

## Materials and methods

### Study subjects and data collection

Our study investigates the impact of enterprises’ engagement in poverty relief on the cost of equity capital in China. We thus consider all Shanghai and Shenzhen Stock Exchange listed companies. We conduct our empirical analysis from 2013 to 2016 and use two databases: the China Stock Market & Accounting Research Database (CSMAR), which provides financial and corporate governance data, and the Research and Set database (RESSET), which offers institutional investor data. The enterprises’ targeted poverty alleviation data come from manually collected listed company annual reports and social responsibility reports, which can also be found in CSMAR. We set the sample period from 2013 to 2016 for the following two reasons: First, Xi Jinping, the chairman of China, first proposed the concept of “Targeted Poverty Alleviation” during an inspection in Xiangxi in 2013. Since then, targeted poverty alleviation has been highly valued. Thus, we use 2013 as the initial year. Second, the accessibility of the cost of equity capital data is limited to 2016. The sample is further processed according to the following steps. First, we exclude companies that are specially treated or particular transferred (ST, PT). Second, we exclude firms whose earnings per share forecasts in the t+2 period (FEPS_i,t+2_) is less than earnings per share forecasts in the t+1 period (FEPS_i,t+1_), which ensures that the estimates of the price-earnings growth model (PEG) have a positive root. Third, we exclude firms with missing research variables. Fourth, we winsorize all continuous variables at the 1% and 99% levels. Finally, we obtain 5600 observations, of which 2180 are for state-owned enterprises and 3420 for private companies. The number of companies that disclosed participation in targeted poverty alleviation was 380. We use Stata13.0 software for data processing.

Table 1 shows the industry and annual distributions of listed companies participating in poverty alleviation efforts in the research sample. From the perspective of industry distribution, listed companies that engage in poverty relief include 15 industries, of which the manufacturing industry has the most samples. From the perspective of annual distribution, the sample distribution from 2013 to 2015 does not vary greatly, but the number has increased significantly, mainly in 2016, which indicates that enterprises’ involvement in poverty alleviation is greatly affected by regulatory agencies.

**Table 1:** Sample distribution.

**Panel A: Sample size by industry**		
**Industry name**	**Observations**	**Proportion (%)**
Agriculture, forestry, animal husbandry and fishery	2	0.53
Mining industry	25	6.58
Manufacturing	204	53.68
Electricity, heat, gas and water production and supply	24	6.32
Construction industry	23	6.05
Wholesale and retail trade	23	6.05
Transportation, warehousing and postal services	20	5.26
Accommodation and catering industry	2	0.53
Information transmission, software and information technology services	14	3.68
Financial industry	16	4.21
Real estate industry	14	3.68
Scientific research and technical services	1	0.26
Water, environmental and public Facilities management	4	1.05
Health and social work	2	0.53
Culture, sports and entertainment	6	1.58
Total samples	380	100
**Panel B: Sample size by year**		
**Year**	**Observations**	**Proportion (%)**
2013	23	6.05
2014	24	6.32
2015	29	7.63
2016	304	80.00
Total samples	380	100

### Measurements

#### Targeted poverty alleviation performance

According to [50], we use a binary dummy variable (fp) as a proxy for targeted poverty alleviation performance; if the company describes participation in poverty alleviation efforts in annual reports or CSR reports, the value of fp is 1 and 0 otherwise. The corporate social responsibility performance of targeted poverty alleviation referred to in this article is its real existence activity, not just the words of the company. The specific information is as follows: First, the company will disclose poverty alleviation information in its annual report, such as investing in poverty alleviation funds to help poor areas rebuild their houses. The anual report information can be queried by listed companies on the Juchao website (Tide Information, http://www.cninfo.com.cn/new/index), a listed company information disclosure website designated by the China Securities Regulatory Commission (CSRC), and it has certain affirmative significance and legal effect; Second, listed companies describe the specific amount and measures of poverty alleviation in the social responsibility report; Finally, the Shanghai Stock Exchange and the Shenzhen Stock Exchange issued relevant regulations in 2016 forcing listed companies to prepare a poverty alleviation table in the “Important Matters” section of the annual report. The investment funds and materials, the amount of industrial poverty alleviation involved, and the amount of ecological poverty alleviation, among other information, are mandatory for inclusion in the targeted poverty alleviation table. Therefore, participation of a listed company in poverty alleviation must be disclosed, whereas a lack of participation in targeted poverty alleviation is not disclosed. As long as it is a listed company that participates in targeted poverty alleviation, it must disclose the total amount of poverty alleviation input, the amount of specific poverty alleviation project investment and whether there is a follow-up poverty reduction plan, etc., and disclose the details of poverty alleviation in the social responsibility report. Therefore, the article considers that if the annual report or social responsibility report describes in detail a listed company’s poverty alleviation funds or materials, the amount of poverty alleviation projects is greater than 0, or the total amount of targeted poverty alleviation investment in the poverty alleviation table in the annual report is greater than 0, then the listed company has participated in targeted poverty alleviation (the value of fp is 1), otherwise it indicates no participation (the value of fp is 0). For example, if the annual report or social responsibility report discloses the investment of the listed company's funds or materials in the poor, the total amount of the poverty alleviation project is greater than 0, or the total amount of the targeted poverty alleviation input in the poverty alleviation form in the annual report is greater than 0, it is believed that it participates in targeted poverty alleviation, and the value of fp is 1; otherwise, fp is 0. In addition, the poverty alleviation data of Chinese listed companies can be found in CSMAR since 2016, whereas previous treatment of CSR performance can be found in Kinder, Lydenberg, Domini and Co. (KLD) or Thomson Reuters data on firm environmental, social, and corporate governance (ESG) [12,32].

#### Cost of equity capital

There are a number of metrics for the cost of equity capital, including ex ante and ex post indicators, such as the indicators calculated by the price-earnings growth model (PEG), the modified price-earnings growth model (MPEG), the Ohlson and Juettner Nauroth model (OJ) and the capital asset pricing model (CAPM). Mao et al. [51] use the data of Chinese listed companies to evaluate the effectiveness of different models of the cost of equity capital and find that the ex ante model is better than the ex post model and the PEG model is more effective than other models. In addition, the PEG model is an indicator widely used in many other countries [52,53,20,26,30]. For example, it is used in the USA market [20], Korean market [18] and an international market (30 countries) [30].Thus, we choose the PEG model to calculate the cost of equity capital and use MPEG and OJ models in the robustness test.

$$R\_PEG_{i,t}=\sqrt{\frac{FEPS_{i,t+2}-FEPS_{i,t+1}}{P_{i,t}}}$$ (1)

$$R\_MPEG_{i,t}=\sqrt{\frac{FEPS_{i,t+2}+MPEG_{i,t}\times DPS_{i,t+1}-FEPS_{i,t+1}}{P_{i,t}}}$$ (2)

$$R\_OJ_{i,t}=A+\sqrt{A^{2}+\frac{FEPS_{i,t+1}}{P_{i,t}}}\times \left[\frac{\left(FEPS_{i,t+2}-FEPS_{i,t+1}\right)}{FEPS_{i,t+1}}-\left(\gamma -1\right)\right]$$ (3)

$$A=\frac{\left(\gamma -1+DPS_{i,t+1}/P_{i,t}\right)}{2}$$ (4)

where FEPS_i, t+2_ is the analyst earnings forecast per share at the end of the t+2 period; FEPS_i, t+1_ is the analyst earnings forecast per share at the end of the t+1 period; P_i, t_ is the stock price of the company at the end of the t period; DPS_i, t+1_ represents the cash dividend forecast per share at the end of the t+1 period; and γ-1 represents the earnings growth rate per share [53].

### Model specifications

After determining the main research variables, model (5) is constructed. The definitions and calculation methods of R_PEG and fp are as described above. To deal with the issue of endogeneity, this study takes the value of the cost of equity capital in the next period as the dependent variable.

$$\begin{array}{c} R\_PEG_{i,t+1}=\partial_{0}+\partial_{1}fp_{i,t}+\partial_{2}G{\mathrm{rowth}}_{i,t}+\partial_{3}B{\mathrm{eta}}_{i,t}+\partial_{4}Size_{i,t}+\partial_{5}Lev_{i,t}+\partial_{6}Turnoever_{i,t}\\ +\partial_{7}B\_M_{i,t}+\partial_{8}ROA_{i,t}+\partial_{9}ABS\_DA+\sum Y\mathrm{ear}+\sum I\mathrm{ndus}try+\varepsilon \end{array}$$ (5)

Considering the possible influence of other factors, we also include several control variables in our model. First, we include the sales revenue growth rate (Growth) calculated as operating income increase divided by operating income at time t-1. Second, we include the market beta (BETA) to control for firms’ systematic risk [20]. Third, we include firm size (Size) as the natural logarithm of total assets at the end of the period. Fourth, we include financial risk (Lev) measured using total liabilities divided by total assets [20,30]. Fifth, we include operating risk (Turnover) as operating income divided by the average value of total assets. Sixth, we include book-to-market ratio (B_M) measured using book value divided by market value [30]. Seventh, we also include profitability (ROA) as net profit divided by total assets at time t. Eighth, we include manipulative accruals (ABS_DA) calculated by the modified Jones model to control for information quality. Finally, we include year (∑Year) and industry dummies (∑Industry) to control for the cross-sectional differences in each year and industry, and the regression analysis is performed at the firm level.

## Empirical results

### Descriptive statistics

Table 2 reports the descriptive statistics of the variables included in model (5), which has 5600 observations. The mean value of the cost of equity capital is 0.0928, and the corresponding standard deviation is 0.0484, which indicates that the sample difference is not large. The mean value of fp is 0.0679, and the standard deviation is 0.2515, indicating that the number of listed companies involved in poverty alleviation is still relatively small, corresponding to only 6.79%.

**Table 2:** Summary of statistics.

Variables	N	Mean	Std	Min	Max
R_PEG	5600	0.0928	0.0484	0.0000	0.2798
fp	5600	0.0679	0.2515	0.0000	1.0000
Growth	5600	0.2259	0.6296	-0.4996	4.6081
Beta	5600	1.0753	0.2438	0.4470	1.6571
Size	5600	22.3352	1.3883	19.8828	27.0667
Lev	5600	0.4424	0.2134	0.0533	0.9250
Turnover	5600	0.6324	0.4483	0.0467	2.6504
B_M	5600	0.4904	0.2547	0.0800	1.1001
ROA	5600	0.0371	0.0482	-0.1410	0.1857
ABS_DA	5600	0.0661	0.0729	0.0009	0.4416

The descriptive statistics of the control variables in Table 2 are as follows. First, the mean (standard deviations) of Growth is 0.2259(0.6296). Second, the mean (standard deviations) of Beta, Lev, and Turnover are 1.0753(0.2438), 0.4424(0.2134), and 0.6324(0.4483), respectively. Third, the mean (standard deviations) of Size, B_M, and ROA are 22.3352(1.3883), 0.4904(0.2547), and 0.0371(0.0482), respectively. Finally, the mean (standard deviation) of ABS_DA is 0.0661(0.0729). In addition, we find that the VIF value of firm size (Size) is largest, 2.53, and much less than 10, indicating that there is no serious multicollinearity problem.

### Regression results

We report the regression results for model (5) in Table 3. Column 1 present the main result. First, we find a negative association between the cost of equity capital and fp at the 1% significance level, supporting hypothesis. The result indicates that poverty alleviation efforts can reduce the cost of equity capital. That is, for investors, the participation of enterprises in targeted poverty relief work is a signal of firm value improvement and risk reduction rather than an agency cost of reducing investment efficiency. As investors perceive reduced corporate risk, they are more likely to accept a lower reward corresponding to the lower risk, that is, a lower cost of equity capital.

**Table 3:** Targeted poverty alleviation social responsibility performance and cost of equity capital.

Variables	(1) R_PEG_i.t+1_	(2) R_***PEG_i.t+1_	(3) R_PEG_i.t+1_	(4) R_PEG_i.t+1_
fp	-0.0077***	-0.0073***		
	(-2.9888)	(-2.8191)		
fpm			-0.1401*	-0.1352*
			(-1.9115)	(-1.8321)
csr		-0.0032*		-0.0035*
		(-1.6847)		(-1.8097)
Growth	0.0005	0.0002	0.0005	0.0003
	(0.4683)	(0.2468)	(0.4959)	(0.2553)
Beta	-0.0065**	-0.0065**	-0.0067**	-0.0067**
	(-2.2699)	(-2.2502)	(-2.3528)	(-2.3273)
Size	-0.0011	-0.0005	-0.0014	-0.0007
	(-1.1680)	(-0.4454)	(-1.4333)	(-0.6213)
Lev	0.0219***	0.0215***	0.0220***	0.0215***
	(4.3613)	(4.2750)	(4.3754)	(4.2822)
Turnover	-0.0011	-0.0010	-0.0012	-0.0011
	(-0.5783)	(-0.5173)	(-0.6334)	(-0.5664)
B_M	0.0482***	0.0474***	0.0484***	0.0475***
	(8.9988)	(8.7855)	(9.0224)	(8.7979)
ROA	-0.0461***	-0.0464***	-0.0449**	-0.0453**
	(-2.6100)	(-2.6311)	(-2.5436)	(-2.5692)
ABS_DA	0.0371***	0.0367***	0.0368***	0.0365***
	(3.9030)	(3.8753)	(3.8819)	(3.8537)
Constant	0.0977***	0.0847***	0.1032***	0.0890***
	(4.7826)	(3.8278)	(5.0914)	(4.0342)
Year	Included	Included	Included	Included
Industry	Included	Included	Included	Included
Observations	5,600	5,600	5,600	5,600
R-squared	0.1524	0.1531	0.1517	0.1524

Note: The results are clustered at the firm level with robust t statistics in parentheses.

***, **, and * indicate significance at the 1%, 5%, and 10% levels, respectively.

In addition, considering that existing research has found that CSR reporting will reduce the cost of equity capital [20], poverty alleviation engagement is also within the scope of CSR. If a firm is involved in targeted poverty alleviation, it will disclose information about it in CSR reporting. Therefore, the reduction of the cost of equity capital may also be caused by the initiation of CSR reporting and not by the fulfillment of targeted poverty alleviation. To eliminate this possibility, we include CSR reporting (csr) in model (5). If CSR reporting is initiated, csr takes a value of 1 and 0 otherwise. Column 2 reports the regression result after controlling CSR reporting. The coefficient of csr is significantly negative, similar to the previous literature [20], and the relation between fp and the cost of equity capital is robust. In addition, we replace CSR reporting at time t with CSR reporting at time t+1, and the (untabulated) result remains the same. Therefore, the main conclusion does not change substantially.

We also consider the investment intensity of targeted poverty alleviation, which may also affect the cost of equity capital if the hypothesis is confirmed. In Column 3, we replace fp with fpm, which is calculated as the disclosed amount of targeted poverty alleviation input divided by the operating income and multiplied by 100 [54]. In Column 4, we include csr reporting (csr) on the basis of column 3. The results in column 3 and column 4 show that the investment intensity of targeted poverty alleviation can reduce the cost of equity capital, which further explains that the poverty alleviation efforts of enterprises can reduce the cost of equity capital, supporting hypothesis.

The regression results for the control variables show that the coefficient of manipulative accruals (ABS_DA) is significantly positive, indicating that the lower the information quality, the higher the cost of equity capital. The coefficient of financial risk (Lev) is significantly positive, indicating that the higher the financial risk, the higher the cost of equity capital. In addition, the coefficient of the book-to-market ratio (B_M) is significantly positive at the 1% level, indicating that the higher the book-to-market ratio, the higher the cost of equity capital.

### Robustness checks

In this section, we report the results of robustness checks. First, we consider different measures of the cost of equity capital. The main test in this paper uses the PEG model to calculate the cost of equity capital. However, the MPEG and OJ models also belong to the ex-ante measurement, which is superior to the ex-post measurement and has been applied by scholars [22,18]. Thus, we use the MPEG model and OJ model to calculate the measures of the cost of equity capital in model (5), and the regression results are shown in Table 4. Column 1 and column 2 report the regression results of the MPEG model and the OJ model, respectively. The coefficients of fp are all significantly negative, consistent with our main result. Additionally, we replace the implementation of targeted poverty alleviation (fp) with the investment intensity of targeted poverty alleviation (fpm) and find that the (untabulated) results remain the same.

**Table 4:** Alternative measurements of the cost of equity capital.

Variables	(1) R_MPEG_i.t+1_	(2) R_OJ_i.t+1_
fp	-0.0067**	-0.0062**
	(-2.4601)	(-2.2299)
Growth	0.0009	0.0005
	(0.6852)	(0.3683)
Beta	-0.0037	-0.0028
	(-1.1987)	(-0.9127)
Size	0.0003	0.0010
	(0.2940)	(0.9111)
Lev	0.0107*	0.0079
	(1.8437)	(1.3638)
Turnover	-0.0021	-0.0019
	(-0.9527)	(-0.9120)
B_M	0.0556***	0.0524***
	(9.0415)	(8.5518)
ROA	0.0418*	0.0168
	(1.7942)	(0.7130)
ABS_DA	0.0261**	0.0256**
	(2.4742)	(2.3997)
Constant	0.0528**	0.0493**
	(2.3534)	(2.1881)
Year	Included	Included
Industry	Included	Included
Observations	4,326	4,193
R-squared	0.1772	0.1736

Note: The results are clustered at the firm level, with robust t statistics in parentheses.

***, **, and * indicate significance at the 1%, 5%, and 10% levels, respectively.

Second, we mitigate the endogeneity issue by running an additional analysis using a change model, where the dependent variable is replaced by the difference between the cost of equity capital in the next period and the cost of equity capital in the current period (ΔR_PEG) and the independent variable is replaced by the difference between the implementation of targeted poverty alleviation in the t period and in the t-1 period (Δfp). The control variables are calculated in change form (i.e., the value for year t minus the value of the same variable for year t-1). The regression results are shown in Table 5. The coefficient of Δfp is still significantly negative, indicating that the change in the implementation of targeted poverty alleviation has a smaller effect on the cost of equity capital, further supporting our hypothesis.

**Table 5:** Change model analysis.

Variables	(1) ΔR_PEG_i.t+1_
Δfp	-0.0070*
	(-1.8291)
ΔGrowth	-0.0007
	(-0.4839)
ΔBeta	-0.0033
	(-0.9336)
ΔSize	0.0085**
	(2.4151)
ΔLev	0.0046
	(0.4276)
ΔTurnover	-0.0004
	(-0.0543)
ΔB_M	-0.0085
	(-0.8537)
ΔROA	-0.0311
	(-0.9828)
ΔABS_DA	-0.0141
	(-1.2777)
Constant	-0.0259***
	(-2.9316)
Year	Included
Industry	Included
Observations	3,299
R-squared	0.1004

Note: The results are clustered at the firm level with robust t statistics in parentheses.

***, **, and * indicate significance at the 1%, 5%, and 10% levels, respectively.

Third, we consider that there may be differences between listed companies that participate in the drive to solve the poverty problem and those that do not, which may lead to self-selection problems in this study. We use the one-to-one propensity score matching method (PSM) to relieve this problem. First, a probit model is used to generate a propensity score according to company characteristics such as firm size, financial risk, profitability and information quality. We also include year and industry dummies in the probit model. Then, we match each company that engages in targeted poverty alleviation with a company that is most similar in terms of firm characteristics but fails to join in poverty alleviation efforts as a control. After matching, the sample is reduced, and finally 760 matching results are obtained, including 380 in the experimental group and the control group. The results are shown in column 1 of Table 6. The coefficient of fp is significantly negative, indicating that the main conclusion remains unchanged, which also means that the result is not seriously affected by sample selection bias and further supports hypothesis.

**Table 6:** Other robustness checks.

	PSM	Excluding financial samples
Variables	(1) R_PEG_i.t+1_	(2) R_PEG_i.t+1_
fp	-0.0059*	-0.0071***
	(-1.7914)	(-2.7081)
Growth	-0.0019	0.0005
	(-1.3264)	(0.5376)
Beta	-0.0055	-0.0072**
	(-0.8063)	(-2.4287)
Size	-0.0027	-0.0011
	(-1.4867)	(-1.0393)
Lev	0.0301**	0.0218***
	(2.0667)	(4.2895)
Turnover	-0.0047	-0.0012
	(-0.9837)	(-0.6192)
B_M	0.0431***	0.0484***
	(3.8213)	(8.9306)
ROA	0.0241	-0.0471***
	(0.4100)	(-2.6581)
ABS_DA	0.0573*	0.0382***
	(1.8119)	(3.9178)
Constant	0.1342***	0.0964***
	(3.0343)	(4.5212)
Year	Included	Included
Industry	Included	Included
Observations	760	5,517
R-squared	0.1871	0.1514

Note: The results are clustered at the firm level with robust t statistics in parentheses.

***, **, and * indicate significance at the 1%, 5%, and 10% levels, respectively.

Fourth, we consider differences in firms between the financial industry and other industries, such as differences in poverty alleviation methods (financial poverty alleviation). We remove the financial industry samples from the full sample and use model (5) to perform an OLS regression. The results are shown in column 2 of Table 6, which has 5517 observations. The coefficient of fp is significantly negative at the 1% level, which is basically consistent with our main conclusion.

### The impact of property nature, region and mandatory disclosure

In this section, we report the results of the further analysis. First, we consider the impact of nature of property. In China, enterprises with different property rights have great differences in organizational behavior. Compared with state-owned enterprises, private enterprises have inherent political weakness [55], and CSR such as charitable donations could establish political relations and function as an important channel for improving political weakness. Under the background of targeted poverty alleviation in China, is there a difference in the relationship between poverty alleviation efforts and the cost of equity capital in enterprises with different property rights? The literature has found that the public has a greater expectation for state-owned enterprises to fulfill their social responsibilities, and investors believe that companies with political connections have lower risks and thus accept lower returns, that is, the cost of equity capital [56]. Thus, we argue that compared with state-owned enterprises, private enterprises are more likely to reduce the risk of enterprises through poverty relief work, and the effect on the cost of equity capital may be more significant. We judge the nature of property rights (SOE) according to the final controller and divide the whole sample into two groups. If the firm is state-owned, the dummy variable SOE takes a value of 1 or 0 otherwise.

The results are shown in column 1 of Table 7. For state-owned enterprises, the coefficient of fp is negative but not significant, whereas the coefficient for the private group is significantly negative at the 1% level, which shows that investors are more sensitive to private enterprises' targeted poverty alleviation engagement and the effect of lowering the cost of equity capital is more significant. In addition, we further divide the state-owned enterprises into central enterprises, provincial enterprises and municipal enterprises and find that the results for different state-owned enterprises are still not significant, which further indicates that the negative relationship between poverty alleviation engagement and the cost of equity capital is significant in private enterprises but not significant in state-owned enterprises.

**Table 7:** The impact of property nature, region and mandatory disclosure.

	(1)Nature of property		(2)Region			(3)Mandatory disclosure	
Variables	State-owned	Private	East	Central	West	2016	Before 2016
fp	-0.0020	-0.0098***	-0.0068**	-0.0108*	-0.0073	-0.0059**	-0.0024
	(-0.5461)	(-2.8584)	(-2.1048)	(-1.8122)	(-1.0758)	(-2.0343)	(-0.4010)
Growth	-0.0040**	0.0010	0.0017	-0.0014	0.0006	0.0018	-0.0000
	(-2.4526)	(0.8330)	(1.4562)	(-0.6209)	(0.2012)	(1.0262)	(-0.0272)
Beta	-0.0054	-0.0042	-0.0097***	0.0101	-0.0139	-0.0042	-0.0085**
	(-1.0602)	(-1.2161)	(-2.8965)	(1.2480)	(-1.6196)	(-0.8800)	(-2.5330)
Size	-0.0021	0.0005	-0.0032***	0.0087***	-0.0007	-0.0051***	0.0001
	(-1.4755)	(0.3896)	(-2.8129)	(3.1101)	(-0.2109)	(-2.9873)	(0.0788)
Lev	0.0348***	0.0154**	0.0212***	0.0203	0.0207	0.0278***	0.0196***
	(4.2461)	(2.4685)	(3.7008)	(1.4206)	(1.4267)	(3.2680)	(3.3995)
Turnover	-0.0036	0.0017	-0.0000	0.0004	-0.0041	-0.0031	-0.0007
	(-1.2076)	(0.7131)	(-0.0102)	(0.0905)	(-0.6370)	(-1.0597)	(-0.3246)
B_M	0.0665***	0.0368***	0.0536***	0.0281**	0.0412***	0.0480***	0.0488***
	(8.1670)	(5.2404)	(8.2372)	(2.1786)	(2.9138)	(4.9955)	(8.2729)
ROA	-0.0165	-0.0606***	-0.0327	-0.0849*	-0.0883*	0.0039	-0.0643***
	(-0.5045)	(-2.8537)	(-1.6177)	(-1.7126)	(-1.7479)	(0.1148)	(-3.1698)
ABS_DA	0.0450***	0.0297**	0.0329***	0.0270	0.0622**	0.0488**	0.0334***
	(2.7746)	(2.5294)	(2.9385)	(1.2261)	(2.1178)	(2.2442)	(3.2100)
Constant	0.0997***	0.0691**	0.1452***	-0.1257**	0.1057*	0.1883***	0.0757***
	(3.4361)	(2.5195)	(6.1135)	(-2.2492)	(1.7306)	(4.8198)	(3.3393)
Year	Included	Included	Included	Included	Included	Included	Included
Industry	Included	Included	Included	Included	Included	Included	Included
Observations	2,180	3,420	3,820	940	760	1,524	4,076
R-squared	0.1956	0.1544	0.1530	0.2152	0.1908	0.1248	0.1526

Note: The results are clustered at the firm level with robust t statistics in parentheses.

***, **, and * indicate significance at the 1%, 5%, and 10% levels, respectively.

Second, since the reform and opening up, the speed of economic development speed has been uneven among various regions in China, in addition to differences in the marketization process, implying great differences in the regional basic environment, such as property rights protection, stakeholder governance, and external supervision. Accordingly, the process by which information is conveyed and interpreted by investors may vary in different regions. We divide the whole sample into three groups, that is, the eastern, central and western regions [57], respectively, to test the impact of enterprises’ poverty alleviation engagement on the cost of equity capital in these three regions, and the results are shown in column 2 of Table 7.

The coefficients of fp in the eastern and central samples are significantly positive at the 5% and 10% levels, respectively, while the corresponding coefficient in the western samples is not significant, indicating that enterprises with better regional economic development have a better signal transmission effect and the negative impact on cost of equity capital is more significant. However, the western region, which has a low marketization process, has not yet established an adequate market system to help investors use the information in the market and thus cannot reflect the signal role of enterprises’ poverty relief work.

Finally, we consider the impact of mandatory disclosure about targeted poverty alleviation on the cost of equity capital. At the end of 2016, the Shanghai and Shenzhen Stock Exchanges issued relevant documents requiring listed companies to disclose specific content in the poverty alleviation table and future plans about poverty alleviation efforts in the “Important Matters” section of the annual report. This requirement may affect the economic consequences of CSR [4]. We distinguish the impact before and after the mandatory disclosure requirement in 2016 and divide the sample into two groups. The results are shown in column 3 of Table 7. After 2016, the coefficient of fp is still significantly negative at the 5% level, while it is not significant before 2016, indicating that the impact on the cost of equity capital mainly exists after the mandatory disclosure requirement in 2016.

### Mechanism test

The above analysis verifies that enterprises’ engagement in poverty relief will reduce the cost of equity capital. Then, what is the underlying mechanism or approach? In the Decision issued in 2016, the China Securities Regulatory Commission noted that institutional investors should be supported and encouraged to join in the battle against poverty. Then, in addition to personally participating in targeted poverty alleviation activities, are institutional investors concerned about the poverty relief work of listed companies? Compared with investors such as retail investors, institutional investors have certain information analysis advantages [58], and existing research has documented that institutional investors do pay more attention to CSR [20,59,60]. For example, institutional investors are attracted by companies with good social responsibility performance and have greater shareholding preference [60]. Institutional investors may regard CSR participation as a signal of the future long-term value of the company and thus increase their shareholding [47]. In addition, some studies have found that institutional investors can reduce information asymmetry and agency costs [61–63,22], which may further reduce the cost of equity capital [22]. Therefore, we believe that enterprises’ involvement in poverty alleviation will further attract institutional investors' attention and increase the shareholding ratio, which will help reduce the information asymmetry and supervision costs of investors, thus reducing the cost of equity capital. We put forward a path to be tested: enterprises’ engagement in poverty relief-the concern of institutional investors-the decline in the cost of equity capital.

Through the mediation effect test [32], we establish three equations of path a, path b, and path c, respectively, and analyze the mediating effect of institutional investors' holdings. The test equations are as follows.

Path a:

$$\begin{array}{l} R\_PEG_{i,t+1}=\partial_{0}+\partial_{1}fp_{i,t}+\partial_{2}G{\mathrm{rowth}}_{i,t}+\partial_{3}B{\mathrm{eta}}_{i,t}+\partial_{4}Size_{i,t}+\partial_{5}Lev_{i,t}\\ \;+\partial_{6}Turnoever_{i,t}+\partial_{7}B\_M_{i,t}+\partial_{8}ROA_{i,t}+\partial_{9}ABS\_DA+\sum Y\mathrm{ear}+\sum I\mathrm{ndus}try+\varepsilon \end{array}$$ (6)

Path b:

$$\begin{array}{l} I\mathrm{ns}\_{\mathrm{share}}_{i,t+1}=\beta_{0}+\beta_{1}fp_{i,t}+\beta_{2}G{\mathrm{rowth}}_{i,t}+\beta_{3}B{\mathrm{eta}}_{i,t}+\beta_{4}Size_{i,t}+\beta_{5}Lev_{i,t}\\ \;+\beta_{6}Turnoever_{i,t}+\beta_{7}B\_M_{i,t}+\beta_{8}ROA_{i,t}+\beta_{9}ABS\_DA+\sum Y\mathrm{ear}+\sum I\mathrm{ndus}try+\varepsilon \end{array}$$ (7)

Path c:

$$\begin{array}{l} R\_PEG_{i,t+1}=\theta_{0}+\theta_{1}fp_{i,t}+\theta_{2}I\mathrm{ns}\_share_{i,t}+\theta_{3}G{\mathrm{rowth}}_{i,t}+\theta_{4}B{\mathrm{eta}}_{i,t}+\theta_{5}Size_{i,t}+\theta_{6}Lev_{i,t}\\ \;+\theta_{7}Turnoever_{i,t}+\theta_{8}B\_M_{i,t}+\theta_{9}ROA_{i,t}+\theta_{10}ABS\_DA+\sum Y\mathrm{ear}+\sum I\mathrm{ndus}try+\varepsilon \end{array}$$ (8)

where Ins_share_i, t+1_ is the proportion of institutional investors shareholding in the next period and the definitions of other variables are the same as above.

We can use the following procedures to judge whether the mediation effect exists. First, if the coefficient (ɑ_1_) of fp in path a is significant, then move to the second step. Second, if the coefficient (β_1_) of fp is significant, it will be transferred to the third step. Third, if the coefficient (θ_2_) of the institutional investor shareholding ratio (Ins_share) in path c is significant, we can conclude that the mediation effect exists. The final judgment is whether it is a partial mediating effect or a complete mediating effect. If the coefficient (θ_2_) of the institutional investor shareholding ratio (Ins_share) is significant and the coefficient (θ_1_) of fp is also significant, then it is a partial mediation effect. However, if θ_1_ is not significant, it is a complete mediating effect. The results are shown in Table 8.

**Table 8:** Mediating effect test.

	(1) path a	(2) path b	(3) path c
Variables	R_PEG _i,t+1_	Ins_share_i,t+1_	R_PEG _i,t+1_
fp	-0.0077***	0.0752***	-0.0071***
	(-2.9888)	(6.4757)	(-2.7552)
Ins_share			-0.0079**
			(-2.5616)
Growth	0.0005	0.0048	0.0005
	(0.4683)	(0.9720)	(0.5084)
Beta	-0.0065**	-0.0038	-0.0065**
	(-2.2699)	(-0.2884)	(-2.2816)
Size	-0.0011	0.0465***	-0.0008
	(-1.1680)	(10.5790)	(-0.7828)
Lev	0.0219***	0.0371	0.0222***
	(4.3613)	(1.6088)	(4.4037)
Turnover	-0.0011	0.0366***	-0.0008
	(-0.5783)	(3.8228)	(-0.4243)
B_M	0.0482***	-0.0494**	0.0478***
	(8.9988)	(-2.1193)	(8.9292)
ROA	-0.0461***	0.1167	-0.0451**
	(-2.6100)	(1.3959)	(-2.5511)
ABS_DA	0.0371***	-0.0332	0.0368***
	(3.9030)	(-0.7702)	(3.8735)
Constant	0.0977***	-0.8360***	0.0910***
	(4.7826)	(-9.3959)	(4.4147)
Year	Included	Included	Included
Industry	Included	Included	Included
Observations	5,600	5,600	5,600
R-squared	0.1524	0.3640	0.1535

Note: The results are clustered at the firm level with robust t statistics in parentheses.

***, **, and * indicate significance at the 1%, 5%, and 10% levels, respectively.

Column 1 of Table 8 reports the result of path a, in which the coefficient of fp(ɑ_1_) is significantly negative at the 1% level, indicating that enterprises’ engagement in poverty relief significantly reduces the cost of equity capital. Column 2 reports the result of path b, in which the fp coefficient (β_1_) is positive and significant at the 1% level, indicating that enterprises’ engagement in poverty relief significantly increases the shareholding ratio of institutional investors. Column 3 reports the result of path c, in which the coefficient (θ_2_) of the institutional investor's shareholding ratio and the coefficient (θ_1_) of fp are both significantly negative at the 1% level. Meanwhile, we find that the Z value in the sober test is 2.28, so it is significant at least at the 5% level, and the 95% confidence interval in the BOOTSTRP test does not include 0, which indicates that the mediation effect is significant. Thus, we believe that the partial mediation effect of the institutional investor shareholding ratio does exist.

In addition, the results of replacing PEG with MPEG and OJ as the cost of equity capital are similar. The result of replacing participation in targeted poverty alleviation with disclosed investment intensity of targeted poverty alleviation does not change substantially, thus further supporting a partial mediation effect. Therefore, the transmission path of “enterprises’ engagement in poverty relief-the concern of institutional investors-the decline in the cost of equity capital” is verified.

### The impact on firm value and financial performance

This study believes that institutional investors have information superiority compared with other investors and can better interpret the valuable signal of corporate targeted poverty alleviation performance, which may indicate increased value of the firm and increase the shareholding ratio. At the same time, institutional investors also play a role in governance supervision. Then, will engagement in targeted poverty alleviation bring about an increase in firm value and financial performance? To test this possibility, we establish models (9) and (10).

$$\begin{array}{c} TobinQ_{i,t}+1=\partial_{0}+\partial_{1}fp_{i,t}+\partial_{2}Size_{i,t}+\partial_{3}Lev_{i,t}+\partial 4Growth_{i,t}+\partial_{5}ROA_{i,t}+\partial_{6}top1_{i,t}+\partial_{7}D{\mathrm{ual}}_{i,t}\\ +\partial_{8}IDP_{i,t}+\partial_{9}SOE_{i,t}+\partial_{10}A{\mathrm{ge}}_{i,t}+\sum Year+\sum Industry+\varepsilon \end{array}$$ (9)

$$\begin{array}{c} ROA_{i,t+1}=\partial_{0}+\partial_{1}fp_{i,t}+\partial_{2}Size_{i,t}+\partial_{3}Lev_{i,t}+\partial_{4}Growth_{i,t}+\partial_{5}ROA_{i,t}+\partial_{6}top1_{i,t}+\partial_{7}D{\mathrm{ual}}_{i,t}\\ +\partial_{8}IDP_{i,t}+\partial_{9}SOE_{i,t}+\partial_{10}A{\mathrm{ge}}_{i,t}+\sum Year+\sum Industry+\varepsilon \end{array}$$ (10)

where TobinQ_i, t+1_ is calculated as the market value divided by the book value of the total assets at time t+1 [64] and ROA_i, t+1_ represents return on assets at time t+1. We also include some control variables. First, we include corporate governance variables [33,64]. We include top1_i, t_ as the proportion of the first major shareholder and a dummy variable Dual that equals 1 if a CEO is also chair of the board and 0 otherwise [33]. We also include IDP as the number of independent outside directors divided by the number of total directors [33]. Second, we control for firm characteristics [33,64], such as Size, Lev, Growth, which are defined above. In addition, we include Age_i, t_ as the listing age of the listing company [64]. Finally, we control for year and industry fixed effects.

The results are shown in Table 9. The coefficient of fp on firm value (TobinQ_i, t+1_) and financial performance (ROA_i, t+1_) is significantly positive, indicating that the firms' targeted poverty alleviation performance can enhance firm value and performance. In turn, institutional investors, who are experts in information analysis, can perceive the prospects of firm development and thus increase the shareholding ratio, which could enhance governance and reduce information asymmetry and consequently decrease the cost of equity capital.

**Table 9:** The impact on firm value and financial performance.

Variables	(1) TobinQ_i.t+1_	(2) ROA_i.t+1_
fp	0.3532***	0.0037**
	(5.1539)	(2.3787)
Size	-0.7230***	0.0011**
	(-19.0637)	(2.2491)
Lev	-0.8847***	-0.0230***
	(-4.0366)	(-6.6841)
Growth	0.1072**	0.0043***
	(2.1253)	(3.0926)
ROA	5.9179***	0.5766***
	(7.3590)	(31.7481)
top1	0.0083***	0.0002***
	(4.3682)	(5.4775)
Dual	0.0574	-0.0009
	(0.9700)	(-0.8228)
IDP	2.5124***	-0.0147*
	(5.1449)	(-1.6993)
SOE	-0.1313*	-0.0046***
	(-1.8511)	(-3.9376)
Age	0.0136	0.0003**
	(1.6125)	(2.1572)
Constant	17.0316***	-0.0111
	(21.1878)	(-1.0127)
Year	Included	Included
Industry	Included	Included
Observations	6,510	6,510
R-squared	0.4636	0.4276

Note: The results are clustered at the firm level with robust t statistics in parentheses.

***, **, and * indicate significance at the 1%, 5%, and 10% levels, respectively.

## Discussion and conclusion

By discussing the relationship between corporate targeted poverty alleviation participation and the cost of equity capital, this study confirms that corporate poverty alleviation work captures the attention of stakeholders and may alleviate corporate financing pressure. This study empirically tests the relationship between targeted poverty alleviation participation and the cost of equity capital of Chinese listed firms while considering the mediating effect of institutional investors' shareholding. The results suggest that targeted poverty alleviation efforts can improve the performance of organizations as manifested in the significant differences between the firm value and financial performance of those companies that engage and do not engage in targeted poverty alleviation activities. A firm that participates in targeted poverty alleviation efforts is highly valued by institutional investors in the market for long-term value improvement, and institutional investors’ attention increases the information transparency and reduces the cost of investor supervision in the capital market, resulting in reduced cost of equity capital for enterprises.

This study contributes to the existing literature on CSR in several ways. First, the study uses institutional investors' shareholding as a mediating factor to explain the link between CSR and the cost of equity capital. The results of the study suggest that institutional investors' shareholding increases with a firm’s targeted poverty alleviation participation. As a result, the information environment and supervision are better for those firms that engage in poverty alleviation work than for firms that do not. Moreover, the findings of this work support the argument that CSR can maximize the wealth of shareholders by reducing deviations from optimal risk taking [10].

Furthermore, the results of this work are intuitive and empirically justify the effects of property nature, region and mandatory disclosure as influencing factors on the relationship between poverty alleviation participation and the cost of equity capital. Specifically, those firms of a private property nature that participate in targeted poverty alleviation lower their cost of equity capital compared to those firms that do not. For firms of a state-owned property nature, it is difficult to conclude that poverty alleviation participation reduces the expected risk. Therefore, this study sheds light on the view that the targeted poverty alleviation efforts of firms can reduce their cost of equity capital in the presence of private property nature. In addition, the differences between regions are similar to those described in a previous study [57]. Due to the uneven development and information environment, the relationship between poverty alleviation participation and the cost of equity capital is not significant in the west region. In contrast, the eastern and central regions are more sensitive to targeted poverty alleviation participation, and in these regions, significant negative associations between targeted poverty alleviation participation and the cost of equity capital are observed. In terms of mandatory disclosure, a key finding is that the negative relationship between targeted poverty alleviation participation and the cost of equity capital is more significant after mandatory disclosure. This finding supports the argument that mandatory disclosure will affect the economic consequences of CSR [4].

Our work proposes some directions for future research. For instance, how does participation in specific poverty alleviation projects, such as education poverty alleviation and health poverty alleviation, affect corporate economic results, such as financing cost? This relationship could be further explored to establish a link between targeted poverty alleviation and corporate economic results. Some interesting questions can also be addressed in exploring such a link. Do other mediating factors drive the negative association between targeted poverty alleviation participation and the cost of equity capital? How do these factors affect it? Addressing these questions and the link between targeted poverty alleviation participation and corporate economic results is a fruitful avenue for further research in this field.
